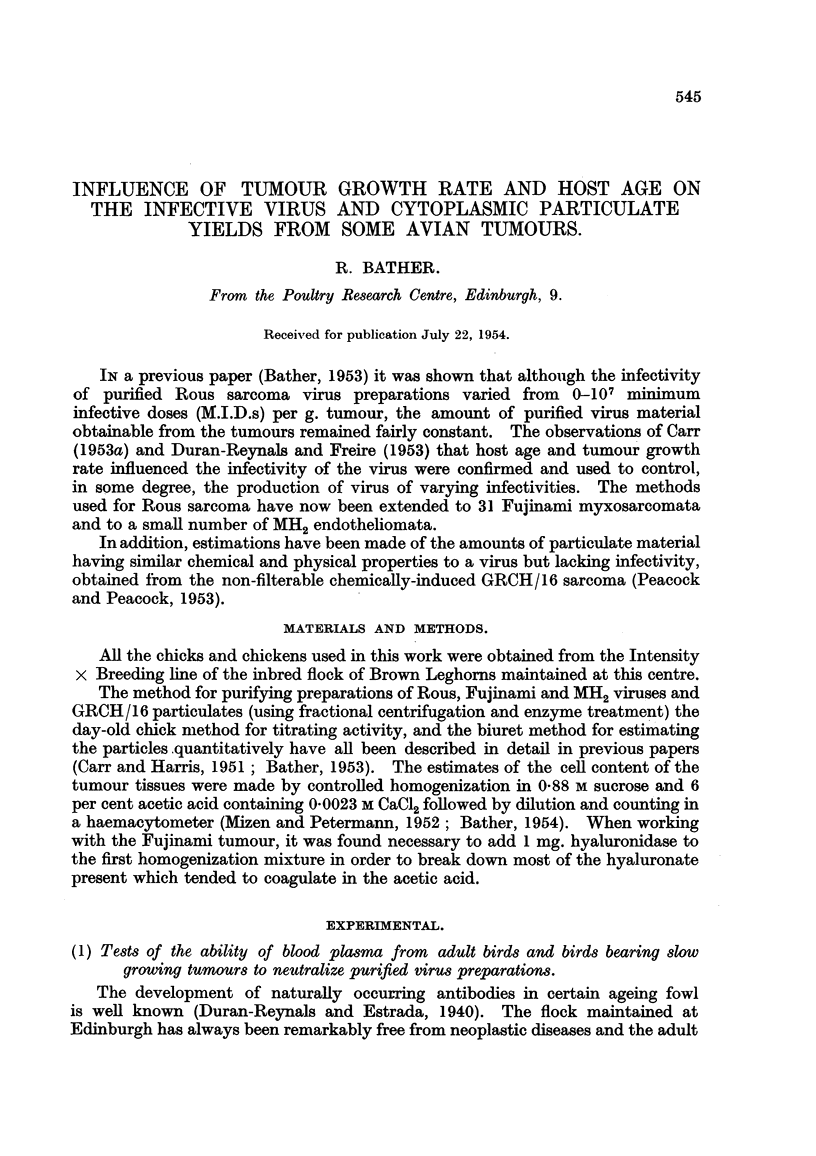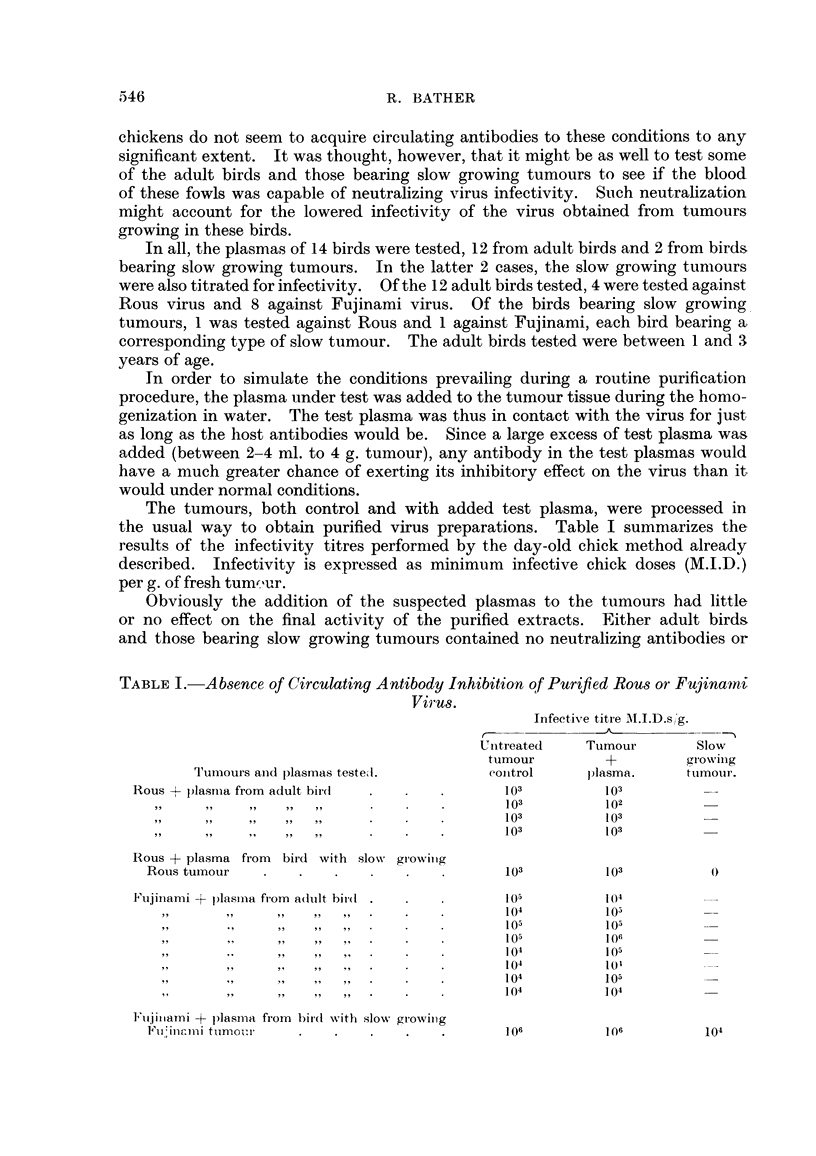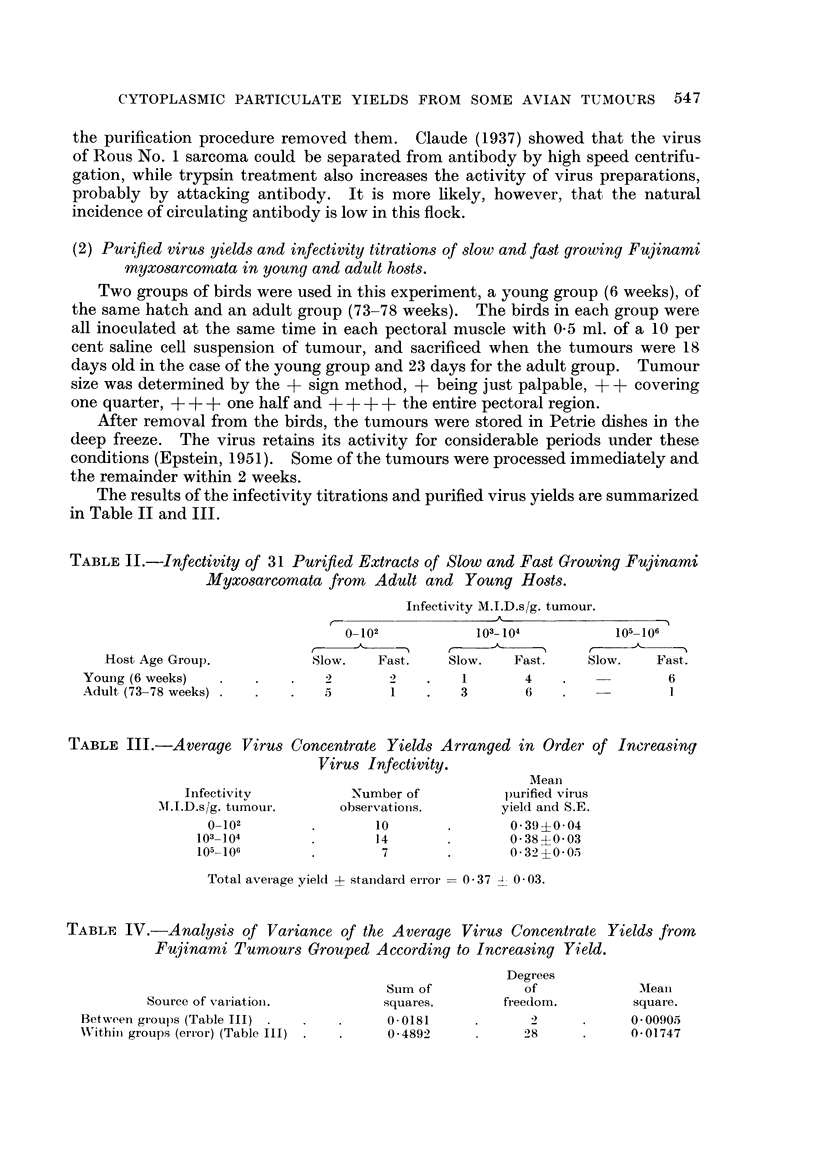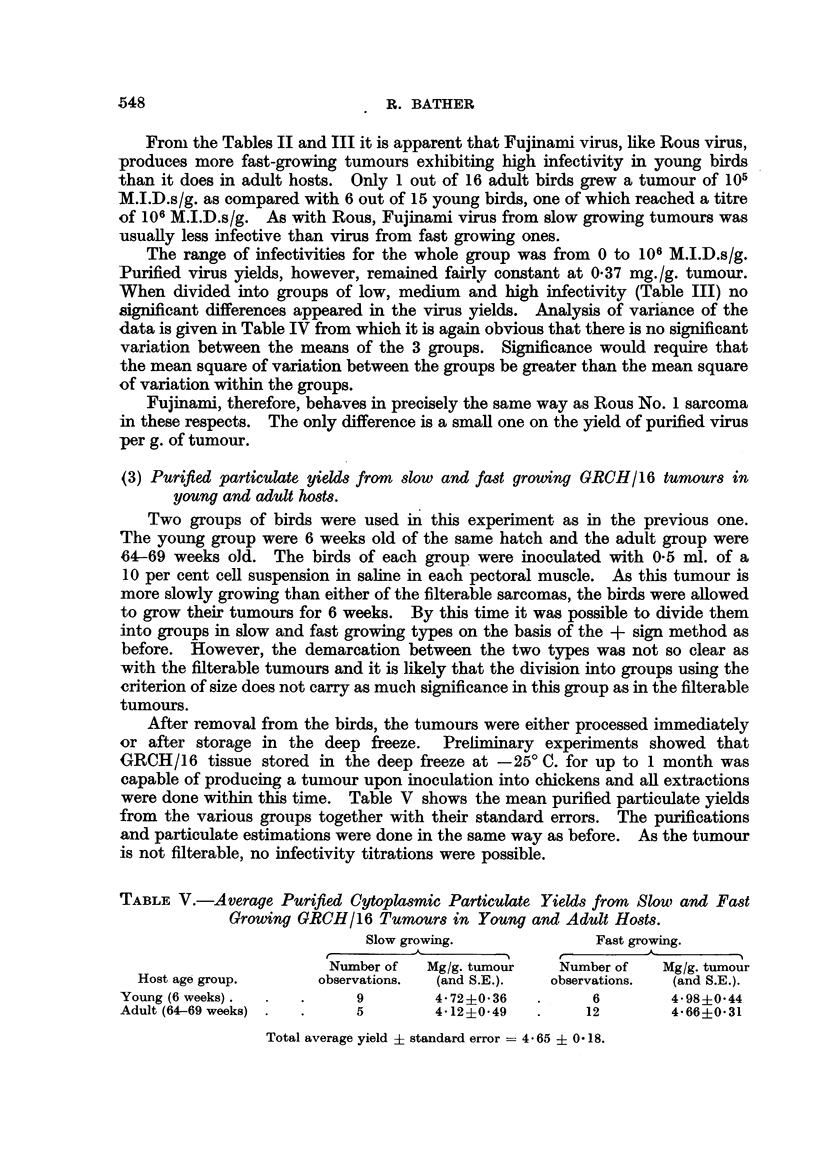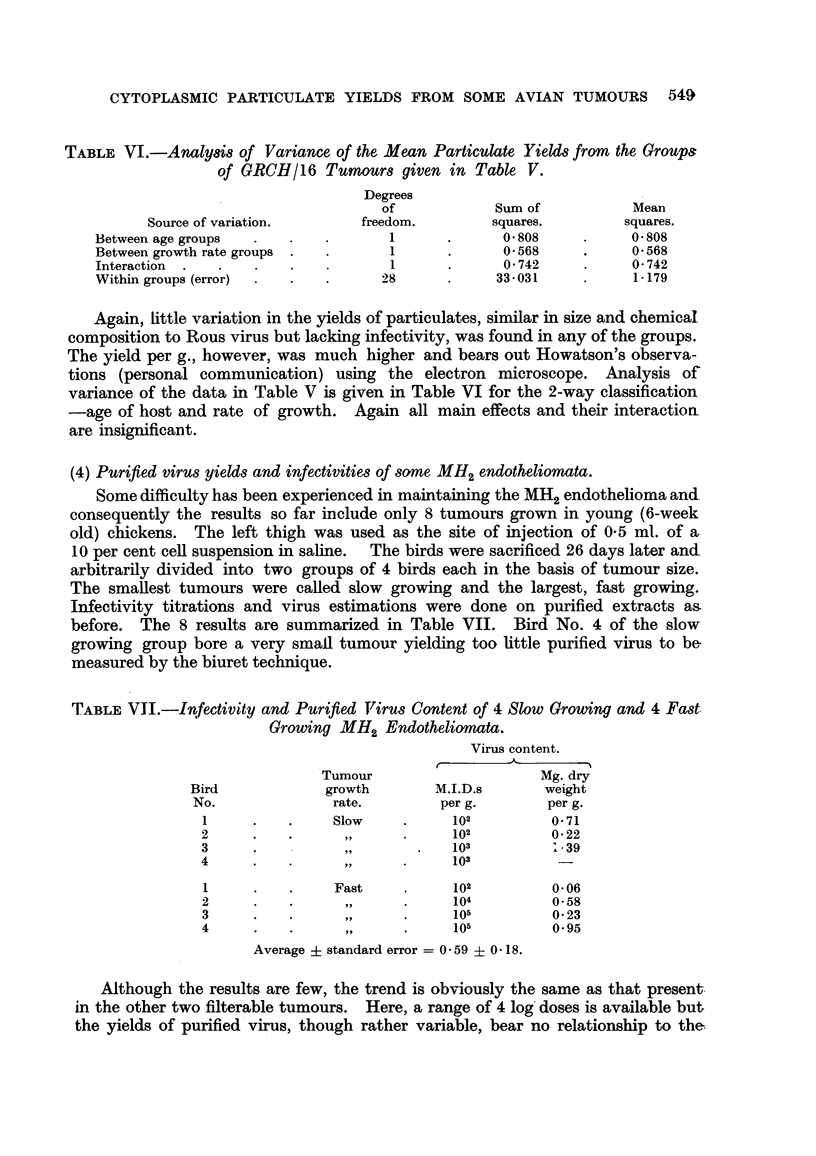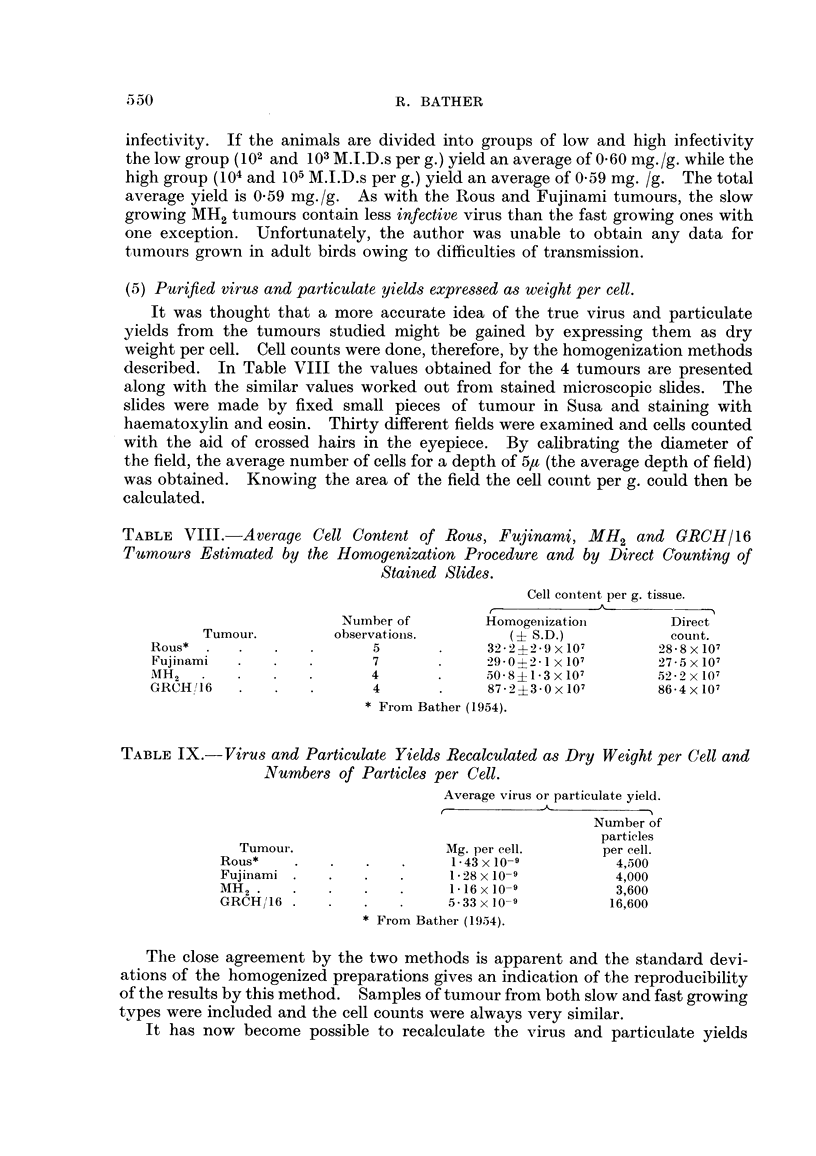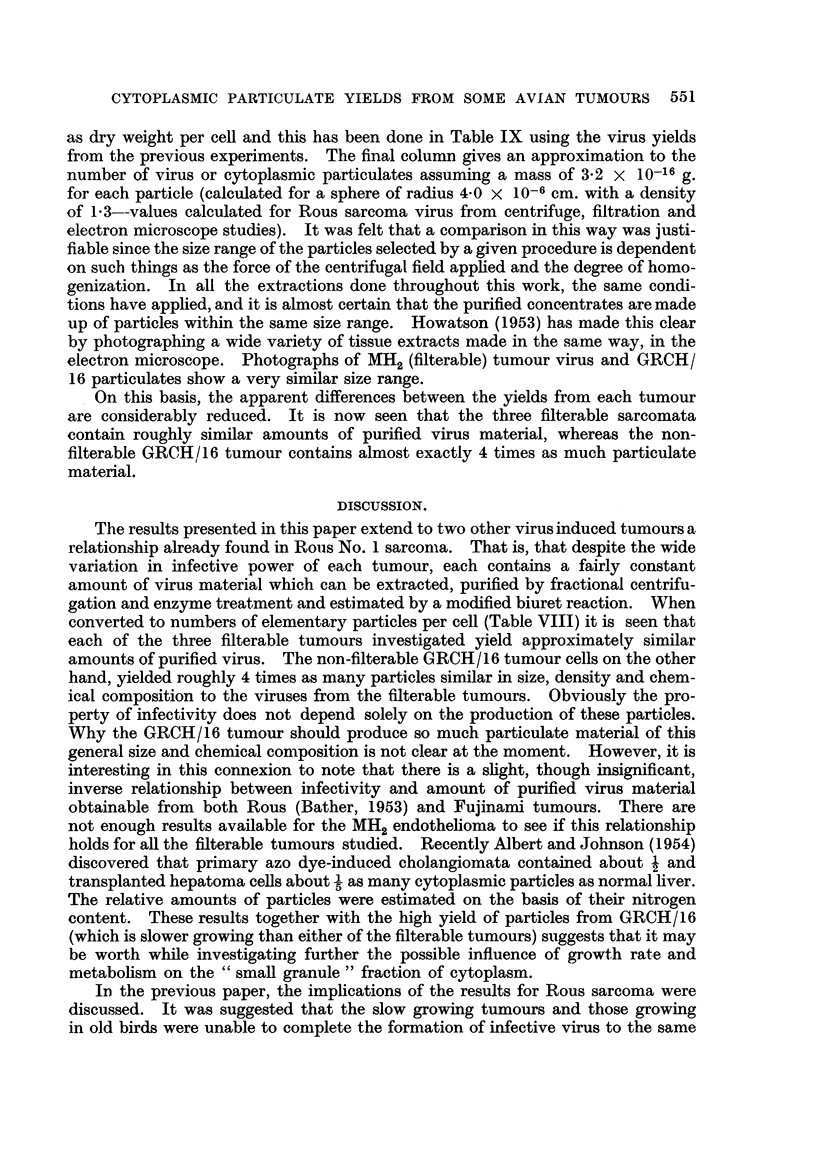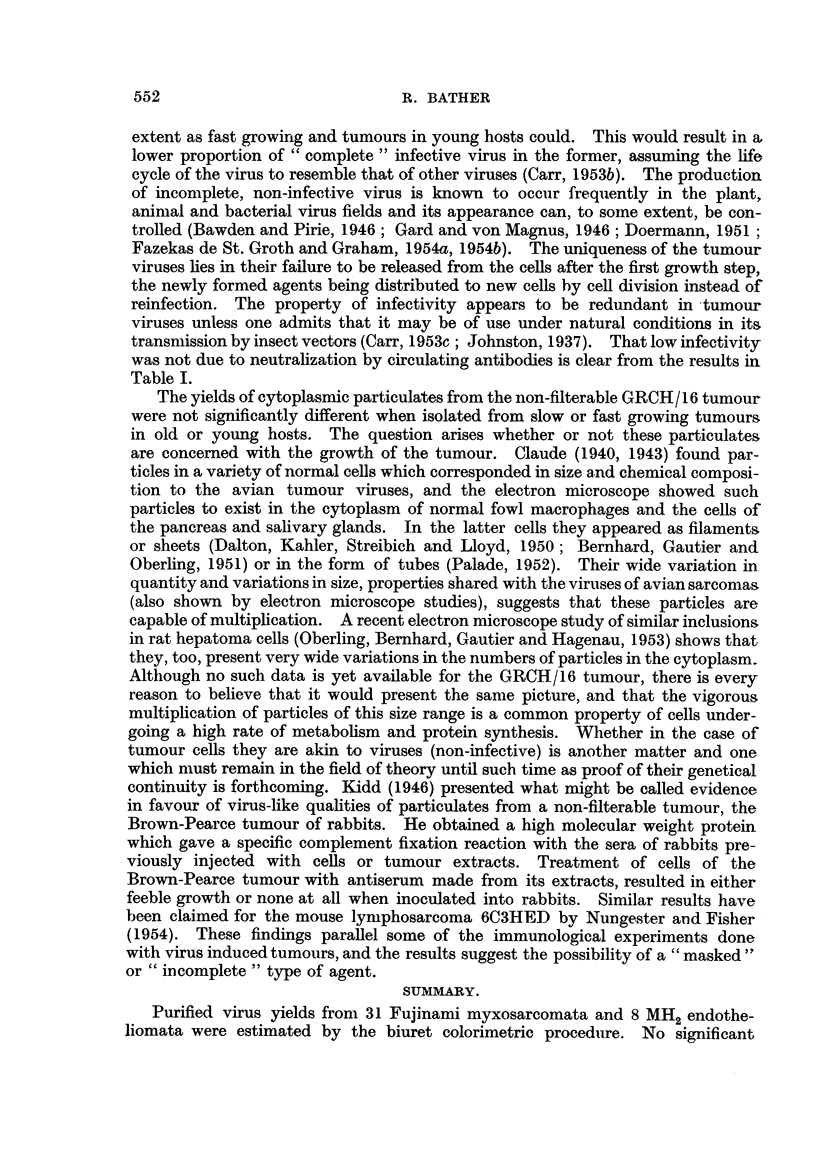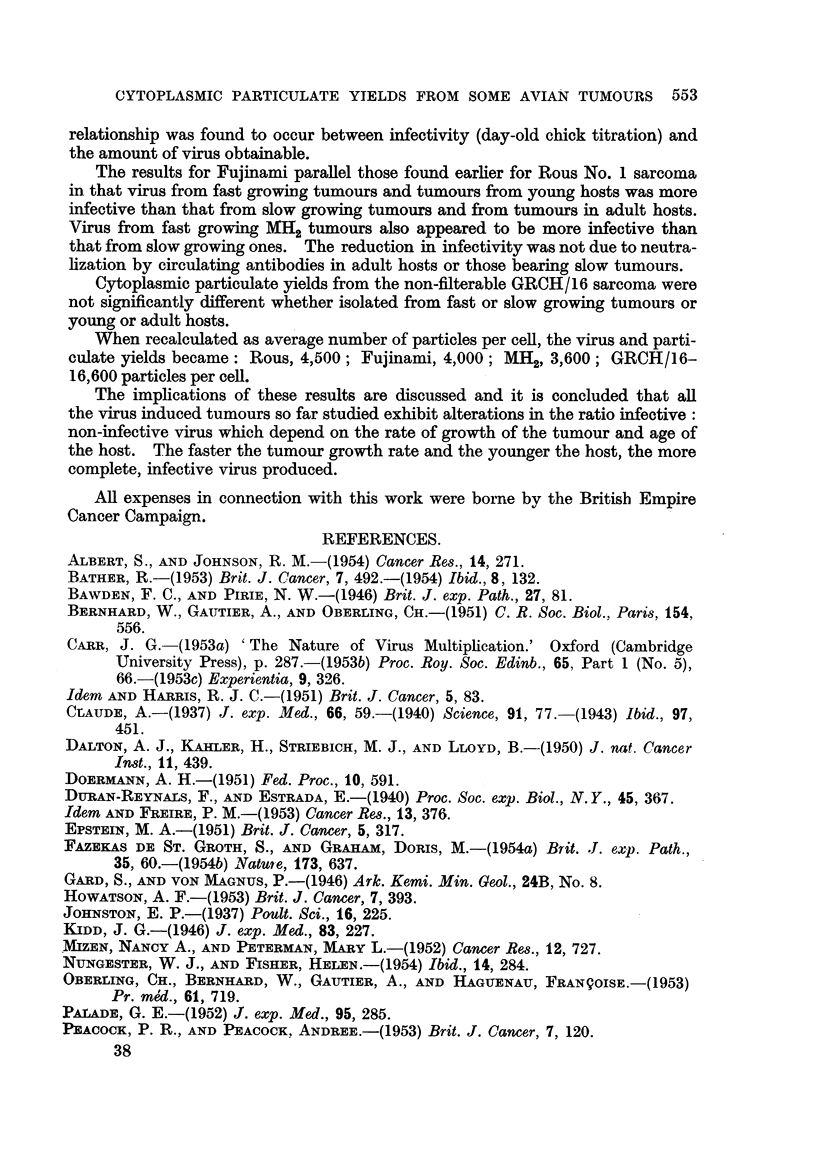# Influence of Tumour Growth Rate and Host Age on the Infective Virus and Cytoplasmic Particulate Yields from some Avian Tumours

**DOI:** 10.1038/bjc.1954.58

**Published:** 1954-09

**Authors:** R. Bather


### INFLUENCE OF TUMOUR GROWTH RATE AND HOST AGE ON THE INFECTIVE VIRUS AND CYTOPLASMIC PARTICULATE YIELDS FROM SOME AVIAN TUMOURS.

R. BATHER.

*From the Poultry Research Centre, Edinburgh, 9.*

Received for publication July 22, 1954.

IN a previous paper (Bather, 1953) it was shown that although the infectivity of purified Rous sarcoma virus preparations varied from 0–$10^7$ minimum infective doses (M.I.D.s) per g. tumour, the amount of purified virus material obtainable from the tumours remained fairly constant. The observations of Carr (1953*a*) and Duran-Reynals and Freire (1953) that host age and tumour growth rate influenced the infectivity of the virus were confirmed and used to control, in some degree, the production of virus of varying infectivities. The methods used for Rous sarcoma have now been extended to 31 Fujinami myxosarcomata and to a small number of $MH_2$ endotheliomata.

In addition, estimations have been made of the amounts of particulate material having similar chemical and physical properties to a virus but lacking infectivity, obtained from the non-filterable chemically-induced GRCH/16 sarcoma (Peacock and Peacock, 1953).

### MATERIALS AND METHODS.

All the chicks and chickens used in this work were obtained from the Intensity × Breeding line of the inbred flock of Brown Leghorns maintained at this centre.

The method for purifying preparations of Rous, Fujinami and $MH_2$ viruses and GRCH/16 particulates (using fractional centrifugation and enzyme treatment) the day-old chick method for titrating activity, and the biuret method for estimating the particles quantitatively have all been described in detail in previous papers (Carr and Harris, 1951 ; Bather, 1953). The estimates of the cell content of the tumour tissues were made by controlled homogenization in 0·88 M sucrose and 6 per cent acetic acid containing 0·0023 M $CaCl_2$ followed by dilution and counting in a haemacytometer (Mizen and Petermann, 1952 ; Bather, 1954). When working with the Fujinami tumour, it was found necessary to add 1 mg. hyaluronidase to the first homogenization mixture in order to break down most of the hyaluronate present which tended to coagulate in the acetic acid.

### EXPERIMENTAL.

### (1) *Tests of the ability of blood plasma from adult birds and birds bearing slow growing tumours to neutralize purified virus preparations.*

The development of naturally occurring antibodies in certain ageing fowl is well known (Duran-Reynals and Estrada, 1940). The flock maintained at Edinburgh has always been remarkably free from neoplastic diseases and the adult chickens do not seem to acquire circulating antibodies to these conditions to any significant extent. It was thought, however, that it might be as well to test some of the adult birds and those bearing slow growing tumours to see if the blood of these fowls was capable of neutralizing virus infectivity. Such neutralization might account for the lowered infectivity of the virus obtained from tumours growing in these birds.

In all, the plasmas of 14 birds were tested, 12 from adult birds and 2 from birds bearing slow growing tumours. In the latter 2 cases, the slow growing tumours were also titrated for infectivity. Of the 12 adult birds tested, 4 were tested against Rous virus and 8 against Fujinami virus. Of the birds bearing slow growing tumours, 1 was tested against Rous and 1 against Fujinami, each bird bearing a corresponding type of slow tumour. The adult birds tested were between 1 and 3 years of age.

In order to simulate the conditions prevailing during a routine purification procedure, the plasma under test was added to the tumour tissue during the homogenization in water. The test plasma was thus in contact with the virus for just as long as the host antibodies would be. Since a large excess of test plasma was added (between 2–4 ml. to 4 g. tumour), any antibody in the test plasmas would have a much greater chance of exerting its inhibitory effect on the virus than it would under normal conditions.

The tumours, both control and with added test plasma, were processed in the usual way to obtain purified virus preparations. Table I summarizes the results of the infectivity titres performed by the day-old chick method already described. Infectivity is expressed as minimum infective chick doses (M.I.D.) per g. of fresh tumour.

Obviously the addition of the suspected plasmas to the tumours had little or no effect on the final activity of the purified extracts. Either adult birds and those bearing slow growing tumours contained no neutralizing antibodies or

TABLE I.—*Absence of Circulating Antibody Inhibition of Purified Rous or Fujinami Virus.*

| Tumours and plasmas tested. | Infective titre M.I.D.s/g. Untreated tumour control | Tumour + plasma. | Slow growing tumour. |
|---|---|---|---|
| Rous + plasma from adult bird | $10^3$ | $10^3$ | — |
| ,, ,, ,, ,, ,, | $10^3$ | $10^2$ | — |
| ,, ,, ,, ,, ,, | $10^3$ | $10^3$ | — |
| ,, ,, ,, ,, ,, | $10^3$ | $10^3$ | — |
| Rous + plasma from bird with slow growing Rous tumour | $10^3$ | $10^3$ | 0 |
| Fujinami + plasma from adult bird | $10^5$ | $10^4$ | — |
| ,, ,, ,, ,, ,, | $10^4$ | $10^5$ | — |
| ,, ,, ,, ,, ,, | $10^5$ | $10^5$ | — |
| ,, ,, ,, ,, ,, | $10^5$ | $10^6$ | — |
| ,, ,, ,, ,, ,, | $10^4$ | $10^5$ | — |
| ,, ,, ,, ,, ,, | $10^4$ | $10^1$ | — |
| ,, ,, ,, ,, ,, | $10^4$ | $10^5$ | — |
| ,, ,, ,, ,, ,, | $10^4$ | $10^4$ | — |
| Fujinami + plasma from bird with slow growing Fujinami tumour | $10^6$ | $10^6$ | $10^4$ |

the purification procedure removed them. Claude (1937) showed that the virus of Rous No. 1 sarcoma could be separated from antibody by high speed centrifugation, while trypsin treatment also increases the activity of virus preparations, probably by attacking antibody. It is more likely, however, that the natural incidence of circulating antibody is low in this flock.

(2) *Purified virus yields and infectivity titrations of slow and fast growing Fujinami myxosarcomata in young and adult hosts.*

Two groups of birds were used in this experiment, a young group (6 weeks), of the same hatch and an adult group (73–78 weeks). The birds in each group were all inoculated at the same time in each pectoral muscle with 0·5 ml. of a 10 per cent saline cell suspension of tumour, and sacrificed when the tumours were 18 days old in the case of the young group and 23 days for the adult group. Tumour size was determined by the + sign method, + being just palpable, ++ covering one quarter, +++ one half and ++++ the entire pectoral region.

After removal from the birds, the tumours were stored in Petrie dishes in the deep freeze. The virus retains its activity for considerable periods under these conditions (Epstein, 1951). Some of the tumours were processed immediately and the remainder within 2 weeks.

The results of the infectivity titrations and purified virus yields are summarized in Table II and III.

TABLE II.—*Infectivity of* 31 *Purified Extracts of Slow and Fast Growing Fujinami Myxosarcomata from Adult and Young Hosts.*

| | Infectivity M.I.D.s/g. tumour. | | | | | |
|---|---|---|---|---|---|---|
| | $0–10^2$ | | $10^3–10^4$ | | $10^5–10^6$ | |
| Host Age Group. | Slow. | Fast. | Slow. | Fast. | Slow. | Fast. |
| Young (6 weeks) | 2 | 2 | 1 | 4 | — | 6 |
| Adult (73–78 weeks) | 5 | 1 | 3 | 6 | — | 1 |

TABLE III.—*Average Virus Concentrate Yields Arranged in Order of Increasing Virus Infectivity.*

| Infectivity M.I.D.s/g. tumour. | Number of observations. | Mean purified virus yield and S.E. |
|---|---|---|
| $0–10^2$ | 10 | 0·39±0·04 |
| $10^3–10^4$ | 14 | 0·38±0·03 |
| $10^5–10^6$ | 7 | 0·32±0·05 |

Total average yield ± standard error = 0·37 ± 0·03.

TABLE IV.—*Analysis of Variance of the Average Virus Concentrate Yields from Fujinami Tumours Grouped According to Increasing Yield.*

| Source of variation. | Sum of squares. | Degrees of freedom. | Mean square. |
|---|---|---|---|
| Between groups (Table III) | 0·0181 | 2 | 0·00905 |
| Within groups (error) (Table III) | 0·4892 | 28 | 0·01747 |

From the Tables II and III it is apparent that Fujinami virus, like Rous virus, produces more fast-growing tumours exhibiting high infectivity in young birds than it does in adult hosts. Only 1 out of 16 adult birds grew a tumour of $10^5$ M.I.D.s/g. as compared with 6 out of 15 young birds, one of which reached a titre of $10^6$ M.I.D.s/g. As with Rous, Fujinami virus from slow growing tumours was usually less infective than virus from fast growing ones.

The range of infectivities for the whole group was from 0 to $10^6$ M.I.D.s/g. Purified virus yields, however, remained fairly constant at 0·37 mg./g. tumour. When divided into groups of low, medium and high infectivity (Table III) no significant differences appeared in the virus yields. Analysis of variance of the data is given in Table IV from which it is again obvious that there is no significant variation between the means of the 3 groups. Significance would require that the mean square of variation between the groups be greater than the mean square of variation within the groups.

Fujinami, therefore, behaves in precisely the same way as Rous No. 1 sarcoma in these respects. The only difference is a small one on the yield of purified virus per g. of tumour.

(3) *Purified particulate yields from slow and fast growing GRCH/*16 *tumours in young and adult hosts.*

Two groups of birds were used in this experiment as in the previous one. The young group were 6 weeks old of the same hatch and the adult group were 64–69 weeks old. The birds of each group were inoculated with 0·5 ml. of a 10 per cent cell suspension in saline in each pectoral muscle. As this tumour is more slowly growing than either of the filterable sarcomas, the birds were allowed to grow their tumours for 6 weeks. By this time it was possible to divide them into groups in slow and fast growing types on the basis of the + sign method as before. However, the demarcation between the two types was not so clear as with the filterable tumours and it is likely that the division into groups using the criterion of size does not carry as much significance in this group as in the filterable tumours.

After removal from the birds, the tumours were either processed immediately or after storage in the deep freeze. Preliminary experiments showed that GRCH/16 tissue stored in the deep freeze at −25° C. for up to 1 month was capable of producing a tumour upon inoculation into chickens and all extractions were done within this time. Table V shows the mean purified particulate yields from the various groups together with their standard errors. The purifications and particulate estimations were done in the same way as before. As the tumour is not filterable, no infectivity titrations were possible.

TABLE V.—*Average Purified Cytoplasmic Particulate Yields from Slow and Fast Growing GRCH/*16 *Tumours in Young and Adult Hosts.*

| Host age group. | Slow growing. Number of observations. | Slow growing. Mg/g. tumour (and S.E.). | Fast growing. Number of observations. | Fast growing. Mg/g. tumour (and S.E.). |
|---|---|---|---|---|
| Young (6 weeks) | 9 | $4 \cdot 72 \pm 0 \cdot 36$ | 6 | $4 \cdot 98 \pm 0 \cdot 44$ |
| Adult (64–69 weeks) | 5 | $4 \cdot 12 \pm 0 \cdot 49$ | 12 | $4 \cdot 66 \pm 0 \cdot 31$ |

Total average yield ± standard error = $4 \cdot 65 \pm 0 \cdot 18$.

TABLE VI.—*Analysis of Variance of the Mean Particulate Yields from the Groups of GRCH/16 Tumours given in Table V.*

| Source of variation. | Degrees of freedom. | Sum of squares. | Mean squares. |
|---|---|---|---|
| Between age groups | 1 | 0·808 | 0·808 |
| Between growth rate groups | 1 | 0·568 | 0·568 |
| Interaction | 1 | 0·742 | 0·742 |
| Within groups (error) | 28 | 33·031 | 1·179 |

Again, little variation in the yields of particulates, similar in size and chemical composition to Rous virus but lacking infectivity, was found in any of the groups. The yield per g., however, was much higher and bears out Howatson's observations (personal communication) using the electron microscope. Analysis of variance of the data in Table V is given in Table VI for the 2-way classification —age of host and rate of growth. Again all main effects and their interaction are insignificant.

(4) *Purified virus yields and infectivities of some $MH_2$ endotheliomata.*

Some difficulty has been experienced in maintaining the $MH_2$ endothelioma and consequently the results so far include only 8 tumours grown in young (6-week old) chickens. The left thigh was used as the site of injection of 0·5 ml. of a 10 per cent cell suspension in saline. The birds were sacrificed 26 days later and arbitrarily divided into two groups of 4 birds each in the basis of tumour size. The smallest tumours were called slow growing and the largest, fast growing. Infectivity titrations and virus estimations were done on purified extracts as before. The 8 results are summarized in Table VII. Bird No. 4 of the slow growing group bore a very small tumour yielding too little purified virus to be measured by the biuret technique.

TABLE VII.—*Infectivity and Purified Virus Content of 4 Slow Growing and 4 Fast Growing $MH_2$ Endotheliomata.*

| Bird No. | Tumour growth rate. | Virus content. M.I.D.s per g. | Virus content. Mg. dry weight per g. |
|---|---|---|---|
| 1 | Slow | $10^2$ | 0·71 |
| 2 | ,, | $10^2$ | 0·22 |
| 3 | ,, | $10^3$ | 1·39 |
| 4 | ,, | $10^3$ | — |
| 1 | Fast | $10^2$ | 0·06 |
| 2 | ,, | $10^4$ | 0·58 |
| 3 | ,, | $10^5$ | 0·23 |
| 4 | ,, | $10^5$ | 0·95 |

Average $\pm$ standard error $= 0·59 \pm 0·18$.

Although the results are few, the trend is obviously the same as that present in the other two filterable tumours. Here, a range of 4 log doses is available but the yields of purified virus, though rather variable, bear no relationship to the infectivity. If the animals are divided into groups of low and high infectivity the low group ($10^2$ and $10^3$ M.I.D.s per g.) yield an average of 0·60 mg./g. while the high group ($10^4$ and $10^5$ M.I.D.s per g.) yield an average of 0·59 mg. /g. The total average yield is 0·59 mg./g. As with the Rous and Fujinami tumours, the slow growing $MH_2$ tumours contain less *infective* virus than the fast growing ones with one exception. Unfortunately, the author was unable to obtain any data for tumours grown in adult birds owing to difficulties of transmission.

(5) *Purified virus and particulate yields expressed as weight per cell.*

It was thought that a more accurate idea of the true virus and particulate yields from the tumours studied might be gained by expressing them as dry weight per cell. Cell counts were done, therefore, by the homogenization methods described. In Table VIII the values obtained for the 4 tumours are presented along with the similar values worked out from stained microscopic slides. The slides were made by fixed small pieces of tumour in Susa and staining with haematoxylin and eosin. Thirty different fields were examined and cells counted with the aid of crossed hairs in the eyepiece. By calibrating the diameter of the field, the average number of cells for a depth of 5$\mu$ (the average depth of field) was obtained. Knowing the area of the field the cell count per g. could then be calculated.

TABLE VIII.—*Average Cell Content of Rous, Fujinami, $MH_2$ and GRCH/16 Tumours Estimated by the Homogenization Procedure and by Direct Counting of Stained Slides.*

| Tumour. | Number of observations. | Cell content per g. tissue. Homogenization (± S.D.) | Direct count. |
|---|---|---|---|
| Rous* | 5 | $32 \cdot 2 \pm 2 \cdot 9 \times 10^7$ | $28 \cdot 8 \times 10^7$ |
| Fujinami | 7 | $29 \cdot 0 \pm 2 \cdot 1 \times 10^7$ | $27 \cdot 5 \times 10^7$ |
| $MH_2$ | 4 | $50 \cdot 8 \pm 1 \cdot 3 \times 10^7$ | $52 \cdot 2 \times 10^7$ |
| GRCH/16 | 4 | $87 \cdot 2 \pm 3 \cdot 0 \times 10^7$ | $86 \cdot 4 \times 10^7$ |

* From Bather (1954).

TABLE IX.—*Virus and Particulate Yields Recalculated as Dry Weight per Cell and Numbers of Particles per Cell.*

| Tumour. | Average virus or particulate yield. Mg. per cell. | Number of particles per cell. |
|---|---|---|
| Rous* | $1 \cdot 43 \times 10^{-9}$ | 4,500 |
| Fujinami | $1 \cdot 28 \times 10^{-9}$ | 4,000 |
| $MH_2$ | $1 \cdot 16 \times 10^{-9}$ | 3,600 |
| GRCH/16 | $5 \cdot 33 \times 10^{-9}$ | 16,600 |

* From Bather (1954).

The close agreement by the two methods is apparent and the standard deviations of the homogenized preparations gives an indication of the reproducibility of the results by this method. Samples of tumour from both slow and fast growing types were included and the cell counts were always very similar.

It has now become possible to recalculate the virus and particulate yields as dry weight per cell and this has been done in Table IX using the virus yields from the previous experiments. The final column gives an approximation to the number of virus or cytoplasmic particulates assuming a mass of $3{\cdot}2 \times 10^{-16}$ g. for each particle (calculated for a sphere of radius $4{\cdot}0 \times 10^{-6}$ cm. with a density of 1·3—values calculated for Rous sarcoma virus from centrifuge, filtration and electron microscope studies). It was felt that a comparison in this way was justifiable since the size range of the particles selected by a given procedure is dependent on such things as the force of the centrifugal field applied and the degree of homogenization. In all the extractions done throughout this work, the same conditions have applied, and it is almost certain that the purified concentrates are made up of particles within the same size range. Howatson (1953) has made this clear by photographing a wide variety of tissue extracts made in the same way, in the electron microscope. Photographs of $MH_2$ (filterable) tumour virus and GRCH/16 particulates show a very similar size range.

On this basis, the apparent differences between the yields from each tumour are considerably reduced. It is now seen that the three filterable sarcomata contain roughly similar amounts of purified virus material, whereas the non-filterable GRCH/16 tumour contains almost exactly 4 times as much particulate material.

### DISCUSSION.

The results presented in this paper extend to two other virus induced tumours a relationship already found in Rous No. 1 sarcoma. That is, that despite the wide variation in infective power of each tumour, each contains a fairly constant amount of virus material which can be extracted, purified by fractional centrifugation and enzyme treatment and estimated by a modified biuret reaction. When converted to numbers of elementary particles per cell (Table VIII) it is seen that each of the three filterable tumours investigated yield approximately similar amounts of purified virus. The non-filterable GRCH/16 tumour cells on the other hand, yielded roughly 4 times as many particles similar in size, density and chemical composition to the viruses from the filterable tumours. Obviously the property of infectivity does not depend solely on the production of these particles. Why the GRCH/16 tumour should produce so much particulate material of this general size and chemical composition is not clear at the moment. However, it is interesting in this connexion to note that there is a slight, though insignificant, inverse relationship between infectivity and amount of purified virus material obtainable from both Rous (Bather, 1953) and Fujinami tumours. There are not enough results available for the $MH_2$ endothelioma to see if this relationship holds for all the filterable tumours studied. Recently Albert and Johnson (1954) discovered that primary azo dye-induced cholangiomata contained about $\frac{1}{2}$ and transplanted hepatoma cells about $\frac{1}{5}$ as many cytoplasmic particles as normal liver. The relative amounts of particles were estimated on the basis of their nitrogen content. These results together with the high yield of particles from GRCH/16 (which is slower growing than either of the filterable tumours) suggests that it may be worth while investigating further the possible influence of growth rate and metabolism on the " small granule " fraction of cytoplasm.

In the previous paper, the implications of the results for Rous sarcoma were discussed. It was suggested that the slow growing tumours and those growing in old birds were unable to complete the formation of infective virus to the same extent as fast growing and tumours in young hosts could. This would result in a lower proportion of " complete " infective virus in the former, assuming the life cycle of the virus to resemble that of other viruses (Carr, 1953*b*). The production of incomplete, non-infective virus is known to occur frequently in the plant, animal and bacterial virus fields and its appearance can, to some extent, be controlled (Bawden and Pirie, 1946 ; Gard and von Magnus, 1946 ; Doermann, 1951 ; Fazekas de St. Groth and Graham, 1954*a*, 1954*b*). The uniqueness of the tumour viruses lies in their failure to be released from the cells after the first growth step, the newly formed agents being distributed to new cells by cell division instead of reinfection. The property of infectivity appears to be redundant in tumour viruses unless one admits that it may be of use under natural conditions in its transmission by insect vectors (Carr, 1953*c* ; Johnston, 1937). That low infectivity was not due to neutralization by circulating antibodies is clear from the results in Table I.

The yields of cytoplasmic particulates from the non-filterable GRCH/16 tumour were not significantly different when isolated from slow or fast growing tumours in old or young hosts. The question arises whether or not these particulates are concerned with the growth of the tumour. Claude (1940, 1943) found particles in a variety of normal cells which corresponded in size and chemical composition to the avian tumour viruses, and the electron microscope showed such particles to exist in the cytoplasm of normal fowl macrophages and the cells of the pancreas and salivary glands. In the latter cells they appeared as filaments or sheets (Dalton, Kahler, Streibich and Lloyd, 1950 ; Bernhard, Gautier and Oberling, 1951) or in the form of tubes (Palade, 1952). Their wide variation in quantity and variations in size, properties shared with the viruses of avian sarcomas (also shown by electron microscope studies), suggests that these particles are capable of multiplication. A recent electron microscope study of similar inclusions in rat hepatoma cells (Oberling, Bernhard, Gautier and Hagenau, 1953) shows that they, too, present very wide variations in the numbers of particles in the cytoplasm. Although no such data is yet available for the GRCH/16 tumour, there is every reason to believe that it would present the same picture, and that the vigorous multiplication of particles of this size range is a common property of cells undergoing a high rate of metabolism and protein synthesis. Whether in the case of tumour cells they are akin to viruses (non-infective) is another matter and one which must remain in the field of theory until such time as proof of their genetical continuity is forthcoming. Kidd (1946) presented what might be called evidence in favour of virus-like qualities of particulates from a non-filterable tumour, the Brown-Pearce tumour of rabbits. He obtained a high molecular weight protein which gave a specific complement fixation reaction with the sera of rabbits previously injected with cells or tumour extracts. Treatment of cells of the Brown-Pearce tumour with antiserum made from its extracts, resulted in either feeble growth or none at all when inoculated into rabbits. Similar results have been claimed for the mouse lymphosarcoma 6C3HED by Nungester and Fisher (1954). These findings parallel some of the immunological experiments done with virus induced tumours, and the results suggest the possibility of a " masked " or " incomplete " type of agent.

### SUMMARY.

Purified virus yields from 31 Fujinami myxosarcomata and 8 $MH_2$ endotheliomata were estimated by the biuret colorimetric procedure. No significant relationship was found to occur between infectivity (day-old chick titration) and the amount of virus obtainable.

The results for Fujinami parallel those found earlier for Rous No. 1 sarcoma in that virus from fast growing tumours and tumours from young hosts was more infective than that from slow growing tumours and from tumours in adult hosts. Virus from fast growing $MH_2$ tumours also appeared to be more infective than that from slow growing ones. The reduction in infectivity was not due to neutralization by circulating antibodies in adult hosts or those bearing slow tumours.

Cytoplasmic particulate yields from the non-filterable GRCH/16 sarcoma were not significantly different whether isolated from fast or slow growing tumours or young or adult hosts.

When recalculated as average number of particles per cell, the virus and particulate yields became : Rous, 4,500 ; Fujinami, 4,000 ; $MH_2$, 3,600 ; GRCH/16–16,600 particles per cell.

The implications of these results are discussed and it is concluded that all the virus induced tumours so far studied exhibit alterations in the ratio infective : non-infective virus which depend on the rate of growth of the tumour and age of the host. The faster the tumour growth rate and the younger the host, the more complete, infective virus produced.

All expenses in connection with this work were borne by the British Empire Cancer Campaign.

### REFERENCES.

ALBERT, S., AND JOHNSON, R. M.—(1954) *Cancer Res.*, **14**, 271.
BATHER, R.—(1953) *Brit. J. Cancer*, **7**, 492.—(1954) *Ibid.*, **8**, 132.
BAWDEN, F. C., AND PIRIE, N. W.—(1946) *Brit. J. exp. Path.*, **27**, 81.
BERNHARD, W., GAUTIER, A., AND OBERLING, CH.—(1951) *C. R. Soc. Biol., Paris*, **154**, 556.
CARR, J. G.—(1953*a*) 'The Nature of Virus Multiplication.' Oxford (Cambridge University Press), p. 287.—(1953*b*) *Proc. Roy. Soc. Edinb.*, **65**, Part 1 (No. 5), 66.—(1953*c*) *Experientia*, **9**, 326.
*Idem* AND HARRIS, R. J. C.—(1951) *Brit. J. Cancer*, **5**, 83.
CLAUDE, A.—(1937) *J. exp. Med.*, **66**, 59.—(1940) *Science*, **91**, 77.—(1943) *Ibid.*, **97**, 451.
DALTON, A. J., KAHLER, H., STRIEBICH, M. J., AND LLOYD, B.—(1950) *J. nat. Cancer Inst.*, **11**, 439.
DOERMANN, A. H.—(1951) *Fed. Proc.*, **10**, 591.
DURAN-REYNALS, F., AND ESTRADA, E.—(1940) *Proc. Soc. exp. Biol., N.Y.*, **45**, 367.
*Idem* AND FREIRE, P. M.—(1953) *Cancer Res.*, **13**, 376.
EPSTEIN, M. A.—(1951) *Brit. J. Cancer*, **5**, 317.
FAZEKAS DE ST. GROTH, S., AND GRAHAM, DORIS, M.—(1954*a*) *Brit. J. exp. Path.*, **35**, 60.—(1954*b*) *Nature*, **173**, 637.
GARD, S., AND VON MAGNUS, P.—(1946) *Ark. Kemi. Min. Geol.*, **24**B, No. 8.
HOWATSON, A. F.—(1953) *Brit. J. Cancer*, **7**, 393.
JOHNSTON, E. P.—(1937) *Poult. Sci.*, **16**, 225.
KIDD, J. G.—(1946) *J. exp. Med.*, **83**, 227.
MIZEN, NANCY A., AND PETERMAN, MARY L.—(1952) *Cancer Res.*, **12**, 727.
NUNGESTER, W. J., AND FISHER, HELEN.—(1954) *Ibid.*, **14**, 284.
OBERLING, CH., BERNHARD, W., GAUTIER, A., AND HAGUENAU, FRANÇOISE.—(1953) *Pr. méd.*, **61**, 719.
PALADE, G. E.—(1952) *J. exp. Med.*, **95**, 285.
PEACOCK, P. R., AND PEACOCK, ANDREE.—(1953) *Brit. J. Cancer*, **7**, 120.